# Expectations of youth victims of violence regarding health care professionals leading them to wellness in South Africa

**DOI:** 10.4102/curationis.v38i2.1547

**Published:** 2015-10-05

**Authors:** Ezihe L. Ahanonu, Firdouza Waggie

**Affiliations:** 1 School of Nursing, University of the Western Cape, South Africa; 2 Interdisciplinary Teaching and Learning Unit, University of the Western Cape, South Africa

## Abstract

**Background:**

Many youth victims of violence report for treatment at the health care facilities in the Western Cape Province of South Africa. It was unclear what the youth expected regarding how they could be led towards wellness by health care professionals following an incident of violence (R1.1).

**Objectives:**

This study sought to explore and describe the expectations of the youth victims of violence with regards to health care professionals (R1.2) leading them to wellness in a selected rural community.

**Method:**

A qualitative, exploratory, descriptive and contextual design was used. Nine focus group discussions were conducted with 58 (23 males, 35 females) purposefully selected youth victims of violence between the ages of 15 and 19. Data analysis was done through open coding. Ethics clearance was received from the University Ethics Committee prior to the study being conducted.

**Results:**

Findings indicated that the youth victims of violence expect the health care professionals (professional nurses, doctors and social workers) working in their community to act as role models, demonstrate a professional attitude, provide health education, provide confidential counselling services, and establish school and community outreach programmes.

**Conclusion:**

This study provides evidence that youth victims of violence have important expectations from health care professionals concerning their wellness. Hence, health care professionals should focus on designing and implementing interventions targeting these expectations.

## Introduction

Youth violence is a global public health challenge which has grim and long-term effects on the physical, mental, and social health of youth (Centers for Disease Control and Prevention 2015:171). Youth violence contributes considerably to the increased incidence of injury, disability and untimely deaths amongst young populations (World Health Organization 2015). Worldwide an estimated 200 000 homicides occur annually amongst youth between the ages of ten and 29 as a result of violence, accounting for 43% of the total number of killings globally each year, and for each youth who is murdered about 20 to 40 more sustain injuries that require medical treatment (World Health Organization 2015).

Violence amongst youth is described as the participation of young people, either as victims or perpetrators, in incidents that involve the threat or use of physical force in the context of interpersonal, inter-communal or other conflict, or crime (Graham, Bruce & Perold 2010:38). Some examples of violent acts common amongst youth include assault, rape or sexual abuse, robbery, threats with weapons, and gang-related violence. It also includes violent behaviour like slapping, or hitting and bullying; these acts, though appearing to be minor, have the propensity to cause more significant emotional distress than physical injury (Centers for Disease Control and Prevention 2015:171; Krug et al. 2002:6–7).

The factors associated with violence amongst youth include drug use, peer pressure (Jewkes et al. 2012), disturbance in social structures predominantly in families, substance abuse and the availability of illegal drugs, such as cocaine and methamphetamine (*tik*), particularly in parts of the Western Cape.

Many studies have established a relationship between the exposure of youth to an incident of violence and the development of problems such as anxiety, depression, post-traumatic stress, aggression, alcoholism, drug and substance abuse, attempted suicides, and risky sexual behaviour (Fowler et al. 2009; McDonald & Richmond 2010). A study conducted by Souverein et al. (2015:18) suggests that youth who were victimised recently are at risk of engaging in antisocial behaviour. There is an increasing interest in youth violence amongst health care professionals due to the grim impact that violence has on the psychological wellbeing of its victims (Butchart 2011; Ruffolo, Andresen & Winn 2013). The established problem of violence amongst young people places a high responsibility on health care professionals to promote wellness amongst youth who have been victims of violence.

## Violence amongst South African youth

Violence has been recognised as a serious health concern in South Africa (National Department of Health 2012:2). For instance, from 2012 to 2013, a total of 31.1 homicides and 355.6 assaults with intent to commit grievous bodily harm per 100 000 population was reported by the South African Police Service (South African Police Service 2013). The 2008 annual report of the National Injury Mortality Surveillance System shows that out of the 31 177 unnatural deaths documented in South Africa in 2008, 9831 (31.5%) occurred as a result of violence. This situation places a heavy burden on health care systems and contributes significantly to health care costs; it also contributes significantly to disability-adjusted life years (Matzopoulos et al. 2008:177–185).

Estimating the national trauma caseload in secondary and tertiary level health care facilities, Matzopoulos et al. (2006:50) report an annual caseload of about 1.5 million trauma cases (40 per 1000 of the population) in South Africa. Amongst young people aged 15 to 34, violence is reported to be the leading cause of unnatural death. The majority of these deaths occurred as a result of sharp object injuries, blunt force injuries, and firearm-related injuries (National Injury Mortality Surveillance System 2010:5–8).

The Western Cape Provincial Department of Health in South Africa has recognised violence amongst the youth as a health priority (Naledi & Househam 2009:641). A survey on risk behaviour amongst youths in the Western Cape in 2010 showed that within the previous six months, about 38 percent of males and eight percent of females had carried a weapon (Reddy et al. 2010). Another study assessing trauma-related admissions during a one-year period, conducted by Nicol et al. (2014) at a major tertiary teaching hospital in Cape Town (a city in the Western Cape), reported high rates of violent interpersonal injury (71.6% of intentional injury). This study further indicated that most of the violence occurred toward males younger than 40 years (74.6); the top three locations where violence-related injuries occurred were on the street (3026 of 6226 [48.6%]), at home (2096 [33.6%]), and at a bar or shebeen (299 [4.8%]).

Since violence amongst the youth is regarded as a health priority in South Africa (Rutherford et al. 2007:764; Seedat et al. 2009:1011) health care professionals have a key role to play in protecting the wellbeing of the youth victims of violence. Nevertheless, it is not clear what this category of youth expect from health care professionals with regards to leading them to wellness. Also, no research study has investigated the expectations of youth victims of violence concerning their wellness, hence the need for this study to be conducted.

## Problem statement

In the communities of the Western Cape, a high number of youth victims of violence report for treatment at the health care facilities, a situation which puts a substantial load on the health care system (Govender et al. 2012:303–306; Western Cape Department of Health n.d.:8). This situation is typical of the health care facilities in the selected rural community in the Theewaterskloof Municipality of the Western Cape where this study was conducted.

Although health care professionals provide treatment for the youth victims of violence when they report to the health care facilities, it was not clear exactly what the youth expect as regards to how they can be led towards wellness by these professionals following an incident of violence. In addition, no research was found that explored and described the expectations of youth victims of violence as to how health care professionals can lead them to wellness in their community.

From the above research problem, a study was conducted to examine the expectations that the youth victims of violence have of health care professionals in terms of leading them to wellness in the community of study.

## Aim of the study

The aim of this study was to explore and describe the expectations of the youth victims of violence with regard to health care professionals leading them towards wellness in the community of study.

The study was conducted as part (phase 1) of a major study with the overall aim to develop a framework which can be utilised by health care professionals in leading youth victims of violence towards wellness in a selected rural community in the Western Cape Province.

## Definition of key concepts

Health care professionals are described as experts involved in rendering therapeutic services to persons and comprise of medical professionals and professional nurses (Forrester & Griffiths 2010:174). In the context of this study, the term health care professionals refers to professional nurses, doctors and social workers.

Lead refers to guide or direct a course. ‘To lead’ in the context of this study refers to leadership, which can be defined as the process whereby a person influences a group of individuals so as to achieve a common goal (Northouse 2010:3). It can be described as a relationship between a leader and his or her followers in which the leader uses power, authority and influence for the creation of a shared vision and the attainment of goals (Jooste & Minnaar 2009:5). In this study, health care professionals are perceived as leaders who have the responsibility to lead youth victims of violence towards attaining wellness (the goal).

Violence is defined as the intentional use of physical force or power, threatened or actual, against oneself, another person, or against a group or community that either results in or has a high likelihood of resulting in injury, death, psychological harm, maldevelopment, or deprivation (Krug et al. 2002:5).

Wellness is defined as: ‘a way of life oriented [*sic*] toward optimal health and well-being in which the body, mind and spirit are integrated by the individual to live life more fully within the human and natural community’ (Myers, Sweeney & Witmer 2000:252).

Youth in this study referred to individuals who are between the ages of 15 and 19.

Youth violence is defined as the involvement of young people, as victims, in incidents that involve the threat or use of physical force in the context of interpersonal, inter-communal, other conflict, or crime. This violence may be perpetrated with or without a weapon and could result in physical injuries or death (Graham et al. 2010:38).

Youth victim of violence in the context of this study refers to a youth who has fallen prey to violence in the community of study and has been involved in one or more random acts of violent behaviour such as physical combat, sexual abuse or rape caused by a person or persons who may be known or not known to the youth.

## Research method and design

### Design

This study employed a qualitative, exploratory, descriptive and contextual design. A qualitative design was followed because it allows an in-depth investigation of a phenomenon through the collection of rich narrative data whilst using a flexible approach to understand and give meaning to the experiences of the participants (Polit & Beck 2006:508). An exploratory research design is generally used in establishing new facts and gathering new information or ideas (Babbie 2010:92). A descriptive research design allows a researcher to describe circumstances and events as they occur naturally (Johnson & Christensen 2012:584) and provides a precise interpretation of situations in order to explain what exists (Burns & Grove 2011:256). Contextual research is used for describing and gaining insight into events in the context of a concrete and natural setting where they occur (Henning, Van Rensburg & Smit 2007:62). This study was contextual in nature because it was limited to the experiences of health care professionals in a rural community in South Africa.

### Population and sampling

According to Christensen, Johnson and Turner (2011:505), a population is ‘the full group of interest to a researcher, to which one wants to generalise and from which the sample is selected’. The target population for this study comprised of youth who had been victims of violence in the community of study.

A purposive, non-probability sampling technique was utilised for the study. Purposive sampling refers to the selection of respondents who will generate the necessary data to meet the objective of a study (Polit & Beck 2012:517).

The respondents who were purposefully selected were high school learners who were between the ages of 15 and 19. They were also residents of the community of study and had been victims of community violence not more than six months prior to partaking in the study**.** For conducting qualitative research studies, Burns and Grove (2011:317) state that the entire number of respondents who participate in a study should be determined by data saturation, which refers to the point when there is no new information being generated. For that reason, in this study the researcher continued with the data collection up until the point of data saturation. A total of nine focus group discussions were conducted among the 58 youth participants.

### Data collection method

Focus group discussion (FGD) was used for data collection in this study because focus group discussion permits a researcher to acquire a broad range of views about a topic from participants in a comfortable non-threatening way and allows the participants to freely express themselves and to clarify their own views. FGDs are also useful in exploring research issues that are unclear, or a new topic about which little is known (Hennink, Hutter & Bailey 2011:136–137).

Nine FGDs were conducted in September 2013. The researcher facilitated the discussions which were conducted in a private and comfortable room. Two pilot FGDs were first conducted to assess whether the issues raised with the participants elicited the necessary information to meet the objective of the study (Liamputtong 2011:6). The results of the pilot FGDs formed part of the overall findings, since they yielded the required information. The FGDs in this study involved between six and eight participants in a group and lasted for a period not more than 60 minutes. The researcher articulated an open-ended question with the purpose of eliciting a discussion on the experiences of the participants. The opening question was followed by relevant probing questions. During the discussions the youth participants were allowed to freely describe their expectations regarding health care professionals leading youth victims of violence to wellness in their community. FGDs continued until data saturation was reached. Field notes were also taken during the discussions giving an in-depth written account of the details and experiences of the researcher during the research process (Given 2008:341).

### Data analysis

Qualitative data analysis is an iterative process of collecting and evaluating the data at the same time with the purpose of maximising the meaning of the data (Creswell 2009:183; Polit & Beck 2012:556). In this study, data analysis involved transcription of the voice recordings of the interviews and writing up of field notes. The steps of Tesch's coding technique (Creswell 2009:186) were used for analysis of the data. Two independent coders analysed the data followed by an inter-coder consensus meeting to reach an agreement about the coding.

### Setting

The study was conducted in a rural community in the Overberg district of the Western Cape. The community is characterised by populations with low levels of socioeconomic status, low levels of education, and high levels of unemployment. In addition, the community faces high levels of violence and drug and substance abuse amongst its youth.

## Ethical considerations

Ethical approval to conduct this study was obtained from the University Senate Research Committee of the Faculty of Community and Health Sciences, University of the Western Cape, South Africa (registration number 13/9/39). Permission was also gained from the relevant authorities and the governing board of the school, where the FGDs were held, provided permission for the study. The ethical principles provided in the *Belmont Report* for guiding research, namely respect for persons, beneficence and justice (National Commission for the Protection of Human Subjects of Biomedical and Behavioral Research 1978), and the International Council of Nurses Code of Ethics for Nurses (International Council of Nurses 2012) directed the ethical approach adopted in this study. All potential participants received information sheets containing the study objectives. Those who consented to participate and were 18 years and older signed a consent form, whilst those younger than 18 were given forms requesting permission from their parents or guardians prior to the study. When the participants assembled for the FGDs, the signed permission forms were collected and the participants also signed an assent form before they were included in the study.

## Trustworthiness

To ensure the trustworthiness of the collected data, Guba and Lincoln's strategies of credibility, transferability, dependability, confirmability and authenticity were applied (Guba & Lincoln 1994; Lincoln & Guba 1985).

## Discussion of findings

### Demographic profile of the youth participants

The demographic profile of the 58 youth participants indicated that they were between the age of 15 and 19. Twenty-three of them were male, whilst 35 were female. Nineteen were grade 9 learners, 18 were grade 10 learners, and 21 were grade 11 learners (see Table 1).

**TABLE 1:** Demographic profile of the youth participants (*n* = 58).

Characteristic	Number (n) of participants
**Age (years)**	
15	4
16	21
17	29
18	3
19	1
**Gender**	
Male	23
Female	35
**Grade level**	
9	19
10	18
11	21

### Expectations of the youth victims of violence

The main theme which arose from the data analysis indicated that the youth victims of violence had important expectations of the health care professionals working in their community in leading them towards wellness. Two categories were identified, namely, building a sound and trusting relationship, and guidance of youth to wellness. Each category and its corresponding sub-categories in combination with the participants’ quotations and embedded literature are discussed. A summary of the findings are presented in Table 2.

**TABLE 2:** Expectations of the youth victims of violence.

Category	Sub-category
Building a sound and trusting relationship	Confidentiality and trust. Information sharing. Professional attitudes. Support.
Guidance of youth victims of violence to wellness	Establishing school programmes. Initiating community outreach programmes. Providing counselling services. Role modelling. xxxxx Providing health education.

### Building a sound and trusting relationship

The participants had an expectation of a sound and trusting relationship with the health care providers working in their community. The following four sub-categories emerged from category 1: confidentiality and trust, support, information sharing, and professional attitudes.

### Confidentiality and trust

Confidentiality relates to the non-disclosure of information relating to an individual without their consent or authorisation, whereas trust is the feeling or assurance that a person can comfortably count on another person (International Council of Nurses 2012). In this study, the youth participants specified that they value confidentiality regarding all private information shared with the health care professionals. For instance, one of the youth participants (a 17-year-old female) stated that they would not want the health care professionals to reveal private information to others who were not involved in their care:

‘They must keep what I said private and I think all of us expect that it should be private.’ (FG9, P2)

Similarly, a 16-year-old male participant articulated the need for respecting personal information:

‘We will appreciate it if they keep our secrets secret and not go telling everyone [*about*] our problems. Everybody has got problems and we must all respect each other so that we don't hurt each other's feelings.’ (FG4, P4)

Another participant (a 17-year-old female) reaffirmed the need for confidentiality from health care professionals:

‘When you tell them your problems, they don't have to tell other people. That is not right, you see. I don't want them to do that.’ (FG7, P1)

It appeared as if the youth participants were uncomfortable disclosing their personal information to health care professionals, probably because they might have had unpleasant experiences with health care professionals in this regard in the past (field notes). This could be the cause of the under-utilisation of available counselling services by youth in the setting of this study.

The provision of confidential services by health care providers for their clients nurtures a relationship characterised by trust and respect. According to the International Council of Nurses (2012), health care providers have an important responsibility to safeguard the privacy of their clients as well, as to maintain confidentiality through the non-disclosure of personal health information. What is more, when health care providers are perceived to be trustworthy by their clients, it is more probable that clients will feel comfortable and willing to provide sensitive information which will contribute to their treatment. Also, they will feel respected.

### Information sharing

Information sharing can be referred to as the transmission of facts, knowledge, details and ideas to persons (Sharma & Petosa 2014). A 17-year-old male participant identified trustworthy information emanating from the health care professionals as being vital for building confidence:

‘Health care professionals are there to give information to us [*youth*] … and help us to be calm and not to be afraid of all that is happening.’ (FG2, P1)

Similarly, accurate and adequate information was expected from the health care professionals, as indicated by a 16-year-old female participant:

‘I expect them to also give us information on the right things and to warn us about the wrong things like drugs and alcohol.’ (FG2, P6)

Effective information sharing in the context of victims of violence could be based on therapeutic communication. Van Servellen (2009:50) defines therapeutic communication as expressing support, providing information and feedback, and correcting distortions whilst providing hope. This type of communication helps clients to trust and collaborate with their health care providers. Therefore, its importance in all phases of client-provider interactions and relationships cannot be over-emphasised (Butts & Rich 2011:281). An 18-year-old participant mentioned that she anticipated the health care professionals to reach out to youth at schools and in the community in general, and to give health-related information about the ways in which they can avoid self-destructive behaviour:

‘I expect them not to only check up on us like taking blood pressure and things like that but I expect them to also come up to us and talk to us about things that has [*sic*] to do with our body, how taking drugs can affect you, things like that because we do that without knowing the effect of it.’ (FG7, P5)

These findings highlight that communication can be an empowering process when health care professionals provide information as a precaution to prevent youth from becoming victims of violence.

### Professional attitudes

Attitude can be described as the way in which a person perceives another person or a situation and which influences the response or behaviour of the individual (Narayana & Rao 2008:29). The youth participants could anticipate that health care professionals exhibited a professional attitude towards them. Sadly, the youth reported that some health care professionals displayed a negative attitude towards them by being rude. A 16-year-old female reported:

‘When you go to the clinic, the nurse is supposed to help you and not be rude to you and say, no, you were here last month, because I go to the clinic and it was what I get there. Here [*the community of study*] they are rude to you. I don't want them to be like that …’ (FG1, P4)

A youth participant (a 17-year-old male) specified that he expected doctors to act according to their role as professionals. He stated that doctors should:

‘… do a better job … Some of them are not so good. Some patients may not be happy with what the doctor did for them.’ (FG9, P4)

Previous studies (Ahanonu 2014; Nalwadda et al. 2011; Wood & Jewkes 2006) conducted in different African countries, including South Africa, have confirmed this finding.

Another youth (an 18-year-old female) indicated that an attitude of ‘can wait’ was reflected by a medical practitioner, which was interpreted as a lack of passion for serving the community:

‘If you go to the doctor and then they say you must come back later as they are on a break. Being on break is not a big thing; if they aren't passionate about their work then it will show.’ (FG9, P5)

It is suggested that health care professionals should treat young people with a professional attitude and provide them with dignified care when they visit the health care facilities. In building a sound and trusting relationship with their clients, health care professionals ought to show a passion and willingness to serve whenever the clinic is open (Small & Small 2011). A true leader has a passion for his or her work and serving other people (Lekalakala-Mokgele 2009:325).

### Support

Rendering support can be defined as offering assistance and reassurance to a person in useful ways (Cambridge University Press 2014b). The youth participants stated that they have profound respect for health care professionals and they reported that they expected them as leaders to be supportive in a compassionate and understanding manner. The perception that health care professionals were supportive leaders who assisted their clients to make informed decisions was mentioned by a 17-year-old female:

‘Like when you [*are*] going through problems, you can go and see a social worker and she will help you … they are always there ready to help…’ (FG9, P1)

It is documented that the provision of support by health care professionals to their clients encourages the development of a sound and trusting relationship between them (Ommen et al. 2011)**.** Support was regarded by the participants as a form of communication that involved talking and listening by both parties. A 17-year-old female stated that health care professionals should provide support to youth, who were victims of violence, soon after a violent experience by listening to them:

‘I think that they can start helping by talking about what we are experiencing … Talking always helps to give relief.’ (FG8, P6)

A 17-year-old male indicated the importance of the health care professionals showing compassion by stating the following:

‘They must also be willing to give a listening ear and listen. When you tell people the right things to do they will want to do the right thing and not make a mess of their lives. They must help us youth to make our lives better …’ (FG8, P4)

### Guidance of youth victims of violence towards wellness

Various approaches for leading youth victims of violence to wellness were proposed by the youth participants. These approaches are discussed under the following sub-categories: establishing school programmes; initiating community outreach programmes; providing counselling services; role modelling; and providing health education.

### Establishing school programmes

Schools are educational organisations of learning that promote the transfer of knowledge and skills to learners aimed at their all-round development using instructional methods (McInerney 2014:23). Schools are also places where the holistic wellbeing (intellectual, physical, psychological, emotional, spiritual and social) of learners should be promoted (Pittman n.d.). The youth participants in this study were of the opinion that health care professionals should be motivated to visit young people at schools, particularly victims of violence, and to provide health services to them. A female participant (a 19-year-old) articulated the view that health care professionals should deliver self-management skills and counselling for youth victims through school programmes:

‘They can come to the school and help the kids that have been physically and mentally abused to give them advice on how to deal with it … I want them to start school programmes and help the youth to get fit and so on and to encourage them and give them advice on how to eat good foods and so on.’ (FG8, P2)

An 18-year-old female pointed out that the school outreach programme would most likely be welcomed by their instructors:

‘They can come to the school and talk to us and help us. The teachers cannot stop them. They [*the youth*] take this dagga [*sic*] in school.’ (FG7, P3)

The youth participants also stated that not only would the youth victims of violence benefit from the school programmes, but all youth learners would benefit from these programmes if they are provided in the school environment. For instance, a 17-year-old male participant said:

‘I will say that even if it is once a week, the doctors and the nurses can come to the school and talk to the whole school and encourage them to eat well and have a healthy lifestyle and not bottle up the problem inside.’ (FG4, P3)

This finding is noteworthy because it agrees with recommendations made by the Centers for Disease Control and Prevention (2015) that all health care professionals should promote the welfare of learners, whilst using an approach referred to as the Coordinated School Health approach which is aimed at improving and strengthening the wellbeing of these young people. The need for these programmes arose from the conviction that like the family unit, the school is an institution accountable for the growth and wellbeing of youth in the community. In addition, it is predicated on the conviction that problems such as violence, substance use and unhealthy lifestyle behaviour, including poor physical fitness, can strongly impact on the positive educational accomplishments of the youth. Therefore, the focus should be on promoting the physical, psychological, intellectual, emotional, social and spiritual wellbeing of youth victims of violence in schools. These victims could be assisted in the avoidance of unhealthy activities, such as substance abuse, and adopting and practising health-promoting behaviour. It is also necessary to mention that the provision of school health services at schools has many advantages that include the creation of opportunities for health education, screening for unreported victims of violence, treatment of minor conditions and ailments, and counselling services for youth learners − especially for the ones who cannot be reached in the community. Thus, it becomes necessary for the government to focus on introducing such services at schools.

### Initiating community outreach programmes

Community outreach programmes can be defined as planned activities aimed at supporting and meeting the needs of community members; mostly persons who are at risk because they might be unable to utilise existing conventional health services (Southard 2010:6). In health service delivery systems, community outreach programmes are usually implemented and coordinated by a multidisciplinary team of health professional, including social workers, community health nurses and doctors, nutritionists, psychologists and occupational therapists. They offer a range of services such as health screening, health education to promote healthy behaviour, outpatient treatment, substance abuse management, counselling and enhancing individual and family support capacities and skills to deal with traumatic life situations like the distress faced by victims of violence. They also may facilitate referral to other resources, such as social and legal services (Hansen-Turton, Miller & Greiner 2009:144; Mullner 2009:607).

The youth participants indicated that they would also want health care professionals to lead the victims of violence towards wellness by conducting community outreach programmes in their community. A 17-year-old female specified that health care professionals can choose to conduct health education during such community outreach programmes:

‘They should go to different places in the community and tell the people about the bad stuffs [*sic*] so that the people can know how they can make themselves well, to be better people and what they can do to be a better person.’ (FG8, P6)

A 16-year-old female indicated that attention should also be paid to meeting the needs of females who had been sexually assaulted in the community:

‘They can start a programme in the community, like with the young girls who have been abused so that they will not do drugs and stuffs [*sic*] like that.’ (FG2, P6)

It was mentioned that a wellness centre could be established in their community and the focus of this centre should be on the youth, particularly on what they could do to foster a healthy lifestyle that leads to wellness. For instance, a 19-year-old female participant stated:

‘They must do the programmes on how you can achieve the wellness. They should organise workshops for the youths on how to achieve wellness.’ (FG8, P2)

### Providing counselling services

Counselling is defined as ‘a professional relationship that empowers diverse individuals, families and groups to accomplish mental health, wellness, education and career goals’ (American Counseling Association 2014:2). It can also be described as a confidential relationship between the trained counsellor and the counselee, which can be on a one-on-one, couple, family, or group basis. Counselling services can be delivered during a single or multiple sessions in different ways, for example by physical face-to-face interaction, telephone, or email (Evans 2013:35–36).

The youth participants in this study were of the opinion that health care professionals should provide counselling for youth who were victims of violence. An 18-year-old female participant said:

‘They can have sessions with us so that we can tell them our problems and experiences and they help can [*sic*] us.’ (FG8, P3)

Another participant (a 19-year-old male) shared the same opinion by disclosing:

‘For me, I think they have a lot to do [*pauses*], talking to us, helping us, guiding and teaching us how to live good lives. You don't need to fight to get respect … you need to show the boys how to be good men.’ (FG9, P6)

### Role modelling

A role model is defined as an individual whose behavioural patterns or accomplishments is or can be emulated by other people, particularly young persons who regard such an individual as a role model (Dictionary.com 2014). Role models are also referred to as guides or mentors. Examples of role models in society include health care professionals and teachers (Burns & Grove 2011). The youth participants indicated that they viewed health care professionals as their role models and they wanted them to be exemplary leaders of a healthy lifestyle that supports wellness. One 16-year-old male participant said that he would like it if health care professionals practice the same wellness behaviour that they tell the youth to practice (role model):

‘… because they are the leaders, they should do what they expect us to do …’ (FG2, P2)

and a 17-year-old male participant stated:

‘They need to lead us by example; show us how to become better people.’ (FG7, P2)

Another participant (an 18-year-old male) also felt that health care professionals should act as role models for the youth in the community:

‘They should show us that they are also doing the things that they want us to do. You cannot tell me not to do something that you know you are doing because it is not right.’ (FG9, P6)

Further, a 17-year-old female stated that health care professionals set a good example for them to emulate:

‘They are setting an example for us. Like doctors and nurses, as they encourage us to do much better, as they help others … we learn to respect each other.’ (FG6, P2)

These findings support the view that role modelling is an important issue for health care professionals in all aspects of practice; whether in the clinical, educational, or research setting. For instance, the Provincial Nursing Strategy of the Western Cape Province states that the leadership capacity of health care professionals, such as nurse managers, should be demonstrated in health care practice (National Department of Health 2009:16).

Chism (2013:48) states that ‘role modelling may be the most subtle form of leadership’ for health care professionals. Therefore, as good role models they should be warm, considerate, knowledgeable, empathic and be able to display self-reflection, emotional intelligence and self-leadership (Feltner et al. 2008; Jooste 2014; O’ Connor 2008). In addition, they should act with integrity and have the ability to communicate well. Communicating well will therefore require of them to be ‘proactive, honest and [*practice*] sensitive communication with clients, relatives and medical staff’ (Philpott & Corrigan 2006:11). It is vitally important for health care professionals to be good role models for their patients, their professional colleagues, and the community in general.

### Providing health education

Health education consists of ‘consciously constructed opportunities for learning involving some form of communication designed to improve health literacy, including improving knowledge and developing life skills, which are conducive to individual and community health’ (World Health Organization 2012:13). It is an element of health promotion that focuses on ‘building individuals’ capacities through educational, motivational, skill-building and consciousness-raising techniques’ (World Health Organization 2012:15).

The youth participants indicated that they needed educational support that would assist them with improving their health and avoiding negative influences, such as violence and substance abuse. For instance one of the participants, a 16-year-old female, described a state of harmony that required health education about the dangers of substance abuse to create a safe environment:

‘I expect them to give us information on the right things and to warn us about the wrong things like drugs and alcohol.’ (FG2, P6)

Another 16-year-old female stated that she expected health care professionals to provide health education on how to avoid dangerous habits and lifestyles:

‘They can provide us with information. Some people like to do the wrong stuff so they should help us set our minds away from the wrong stuffs [*sic*] and lead us in the right direction.’ (FG2, P6)

Furthermore, a youth participant (a 16-year-old male) said:

‘I want them to give me advice when I need it and guide me in the right way. I want them to tell me how to be healthy … They should tell the people in the community to live in peace with each other and stop fighting, say; they should stop taking tik because the effect of it is not good for their lives …’ (FG4, P4)

Health education targeting young people can be implemented in the form of lectures, seminars, workshops and printed materials, such as hand bills or fliers. Generally, it should entail the setting of goals and/or objectives for learning and providing information and materials in order to build the client's confidence and to assist the client to adapt to the prescribed behaviour. According to Tate (cited in Mohanna et al. 2011:20), good interpersonal communication skills are needed for the process of health education with clients.

## Recommendations

There is a need for increased commitment amongst all health care professionals, who provide community health services, to demonstrate their leadership roles in leading youth victims of violence to wellness in the community as the youth in this study reported that they expected them to be good role models and demonstrate their leadership capabilities. Community outreach programmes should be organised by health care professionals for both in-school and out-of-school youth in the community. Programmes can focus on violence prevention, screening for violence-related and other health issues, and health education on ways to promote a healthy lifestyle. It is also necessary that the government focuses on introducing health services at schools.

## Conclusion

Given that the youth victims of violence view health care professionals as leaders, it is crucial that nurses and other health care professionals become effective and participative leaders in order to validate their leadership roles and responsibilities, not only to their clients but also to their professions. Attention should also be paid to providing treatment to those who have experienced violence and community interventions should be designed to reduce violence at home and at school. All of these are critical for improving the wellness of the youth in South Africa.

## Limitation of the study

A key limitation of this study is concerned with the generalisability of the findings. The study was conducted amongst youth victims of violence who were high school learners in the community of study. Out-of-school youth victims, who may possibly have dissimilar viewpoints or experiences when compared with the in-school youth victims of violence, were not included in the study.
